# Completeness, consistency, and duplicity of self-mutilation reports by adolescents in the Notifiable Diseases Information System: evaluation study, Santa Catarina, 2014-2023

**DOI:** 10.1590/S2237-96222026v35e20250412.en

**Published:** 2025-11-28

**Authors:** Thayse de Paula Pinheiro, Deise Warmling, Doroteia Aparecida Hofelmann, Elza Berger Salema Coelho

**Affiliations:** 1 Universidade Federal de Santa Catarina, Faculdade de Saúde Pública,, Florianópolis, SC, Brazil; 2 Universidade Federal do Paraná, Curitiba, PR, Brazil

**Keywords:** Self Mutilation, Adolescent, Notification, Notifiable Diseases Information System, Evaluation Study, Automutilación, Adolescente, Notificación, Sistema de Información de Enfermedades de Notificación Obligatoria, Estudio de Evaluación

## Abstract

**Objective:**

To evaluate the completeness, consistency, and duplicity of self-mutilation reports among adolescents in Santa Catarina, from 2014 to 2023.

**Methods:**

This study evaluated reports recorded in the Notifiable Diseases Information System (*Sistema de Informação de Agravo de Notificação* - SINAN). Completeness was assessed for 18 variables and classified as excellent (≥95.0%), good (90.0%–95.0%), fair (70.0%–90.0%), poor (50.0%–70.0%), and very poor (<50.0%). The consistency of seven variables was analyzed and classified into three categories: excellent (≥90.0%), fair (70.0%–89.0%), and low (<70.0%). Duplicity was checked using the variable “NDUPLIC_N” and by manual verification. Temporal trends were analyzed using Prais–Winsten regression, which classified them as stable, increasing, or decreasing.

**Results:**

A total of 1,403 self-mutilation reports were identified. Completeness was excellent for 12 variables and good for three. Sexual orientation (75.8%) and gender identity (76.2%) were rated fair, while motivation of violence (46.1%) was rated very poor. An increasing trend in completeness was observed for three variables. Consistency was excellent for six variables and fair for one, with an increasing trend in “sex of the person attended versus sex of the perpetrator,” a decreasing trend in “presence of disability or disorder vs. type,” and stability for the others. No duplicate records were found.

**Conclusion:**

The analyzed data showed good quality in the recording of self-mutilation among adolescents in SINAN of Santa Catarina. Strategies such as continuing education for health professionals and revisions to the reporting form are crucial for enhancing data quality and strengthening the planning of interventions to address this form of violence.

## Resumen

**Objetivo:**

Evaluar la completitud, consistencia y duplicidad de los reportes de automutilación por adolescentes en Santa Catarina, de 2014 a 2023.

**Métodos:**

Se trató de un estudio de evaluación de los reportes del Sistema de Información de Enfermedades de Notificación Obligatoria (*Sistema de Informação de Agravo de Notificação* - SINAN). Se analizó la completitud de 18 variables y se clasificó como excelente (≥95,0%), buena (90,0%-95,0%), regular (70,0%-90,0%), deficiente (50,0%-70,0%) y muy deficiente (<50,0%). La consistencia de siete variables se analizó y clasificó como: excelente (≥90,0%), regular (70,0%-89,0%) y baja consistencia (<70,0%). Se verificó la duplicidad mediante la variable “NDUPLIC_N” y por revisión manual. Se analizó la tendencia temporal mediante regresión de Prais-Winsten, clasificándola como estabilidad, aumento o disminución.

**Resultados:**

Se identificaron 1.403 reportes de automutilación. La completitud fue excelente en 12 variables y buena en tres. La orientación sexual (75,8%) y la identidad de género (76,2%) fueron evaluadas como regulares, y la motivación de la violencia (46,1%) como muy deficiente. Tres variables mostraron tendencia de aumento de completitud. La consistencia fue excelente en seis variables y regular en una, con tendencia de aumento en el registro de “sexo de la persona atendida versus sexo del autor”, disminución en “presencia de discapacidad o trastorno vs. tipo” y estabilidad en las demás. No se observaron duplicidades.

**Conclusión:**

Los datos analizados mostraron buena calidad en el registro de automutilación por adolescentes en el SINAN de Santa Catarina. Estrategias como la educación continua de los profesionales de salud y modificaciones en el formulario de notificación son esenciales para mejorar la calidad de los registros y para planificar acciones de enfrentamiento de esta violencia.

**Ethical aspects**

This research respected ethical principles, having obtained the following approval data:

Research ethics committee: Universidade Federal de Santa Catarina

Opinion number: 6,775,433

Approval date: 19/4/2024

Certificate of submission for ethical appraisal: 76226423.8.0000.0121

Informed consent form: Exempt.

## Introduction 

Self-mutilation is characterized by self-inflicted injuries without the intent to end one’s own life (1). The severity can vary according to the frequency and intensity of the injuries, being classified as mild, moderate, or severe (1). The practice of self-mutilation usually begins during adolescence. It is considered a factor that negatively impacts development during this transitional stage from childhood to adulthood, marked by bodily, cognitive, affective, social, and behavioral changes (2). 

A prevalence of self-mutilation of 17.6% was identified among adolescents from 17 countries located in North America, Asia, Europe, and Oceania (3). In Norway, between 2017 and 2018, the rate in a sample of 37,268 adolescents was 16.1% (4). In Thailand, in 2020, among 1,457 adolescents, 5.4% had engaged in self-mutilation (5). In 2021, among 1,180 students in China, a prevalence of 24.0% was observed (6). The variation in global prevalence of self-mutilation may be attributed to diverse factors, such as sociocultural aspects (family and social relationships; and residential region) or methodological aspects (type of sampling, community-based or clinical; time frame; and sex of participants) (3).

In Brazil, the occurrence of self-mutilation also shows variations. In 2018, among 517 students aged 10–14 years in Minas Gerais, the proportion of self-mutilation was 9.5%, with five or more occurrences in 2016 (7). In 2017, a slightly lower prevalence of 6.5% was identified in households of 505 adolescents aged 12–17 years from Maceió (8). On the other hand, in 2019, a considerably higher prevalence was observed in a study conducted with 878 students from inland municipalities of Rio Grande do Sul, where 53.0% of participants reported self-mutilation (9). The study highlighted factors that may have influenced these results, including social contagion, stigma, and a lack of privacy in smaller communities, as well as access to the internet and social media, and the scarcity of recreational activities (9).

The prevalence and characteristics of self-mutilation can be verified in the Notifiable Diseases Information System (*Sistema de Informação de Agravos de Notificação* - SINAN). In 2014, the report instrument was modified, being renamed the Report/Investigation Form for Interpersonal/Self-Inflicted Violence, which increased attention to self-inflicted violence (10). Suicide attempts became subject to immediate report (within 24 hours) at the municipal level, aiming to ensure appropriate intervention for the cases (10). Self-mutilation should be reported by all health professionals, preferably by the one who provided care (11), as it is relevant for identification, referral, and prevention of self-mutilation cases.

To strengthen the surveillance system for this condition, it is essential to have a high-quality database with complete, accurate, and original data, free from duplicate records, and with consistently filled fields (12). Similar national studies were found in Santa Catarina (13,14), but focused on analyzing the completion of sexual violence reports. In Niterói, the degree of completeness of reports of violence against older adults was analyzed (15), and in Mato Grosso do Sul, completeness and consistency were analyzed, focusing on indigenous women (16). 

SINAN is the main tool for recording self-mutilation in the health sector in Brazil (10). To the best of our knowledge, no previous Brazilian study has addressed the quality of self-mutilation records, making this work both relevant and innovative. The results may support actions at the three levels of government, aiming to improve the quality of data related to this type of self-inflicted violence. 

This study aimed to evaluate the completeness, consistency, and absence of duplicate records in self-mutilation reports among adolescents in the Notifiable Diseases Information System (SINAN) in Santa Catarina, from 2014 to 2023.

## Methods 

### Study design and setting

This was a normative evaluation study on the quality of self-mutilation reports among adolescents recorded in the Notifiable Diseases Information System (SINAN) (17). Santa Catarina is located in the southern region of Brazil, with an estimated population of 8,058,441 inhabitants in 2024 (18). According to projections, in 2025, the adolescent population in the state is expected to be 1,032,531, comprising 531,693 males and 500,838 females (19).

The initial year of the study was 2014, when the data collection instrument was updated and renamed the Report/Investigation Form for Interpersonal/Self-Inflicted Violence, which increased attention to self-inflicted violence (20). In this updated form, the completion instructions clearly specify the need to identify the type of self-inflicted violence (10). The final year of the study, 2023, was determined by the availability of data at the time of the research.

### Participants 

Reports of self-mutilation among adolescents (10–19 years) were included within the records of self-inflicted violence. The definition of self-mutilation adopted follows that established by the Brazilian Ministry of Health, characterized as an action in which a person deliberately inflicts harm on themselves, typically through cutting or burning, without suicidal intent (21). The age range used in the Surveillance of Violence and Accidents aligns with that of the World Health Organization (10). 

To identify self-mutilation reports, according to the SINAN completion instructions, when field 54 was filled with “1 – Yes” for self-inflicted violence, field 56 had to be completed with “1 – Yes” in the “Other” box. The self-inflicted violence also had to be described (indicating whether it referred to self-mutilation or a suicide attempt) (10). This was an open-text field requiring the information to be written in full (21). Only reports in which the “Other” field was completed as self-mutilation were included. Records left blank, referring to suicide attempts, or insufficient to identify the type of self-inflicted violence, were excluded. This strategy aimed to minimize selection bias and ensure consistency among the analyzed cases.

### Variables 

The quality of the records was assessed in terms of duplicate records, completeness, and consistency. The reporting form variable “NDUPLIC_N” was examined for the analysis of duplicate records.

Regarding completeness, the following mandatory fields were evaluated: sex; sexual orientation; gender identity; municipality of occurrence; place of occurrence; motivation of the violence; relationship/degree of kinship with the victim; sex of the probable perpetrator; and life stage of the probable perpetrator (10–19 years). Essential fields included: race/skin color; education; marital status; recurrence of violence (whether it occurred multiple times); suspected alcohol use; number of individuals involved; and three means of aggression (sharp object, blunt object, and hot substance/object). (12). Failure to complete a mandatory field prevented the report from being included in SINAN. An essential field was defined as one that recorded information necessary for case investigation or for calculating epidemiological or operational indicators (10). 

The completeness of the records referred to the filling of fields in the reporting form, each with a specific purpose aimed at characterizing the violence, analyzing the situation, and implementing preventive measures (12). Completeness was measured based on the proportion of records with ignored or blank fields, classified into five categories: excellent (≥95.0%), good (90.0%–95.0%), fair (70.0%–90.0%), poor (50.0%–70.0%), and very poor (<50.0%) (12). Fields marked as ignored (number 9) or left blank (missing) were considered incomplete. 

The evaluation of consistency aimed to detect incorrectly filled data in the reporting forms and to allow for subsequent correction (12). Consistency was determined by the proportion of related variables that had coherent values without contradictions and was classified into three levels: excellent (coherence ≥90.0%), fair (70.0%–89.0%), and low consistency (<70.0%). 

The percentage of inconsistency was calculated by dividing the number of reporting forms with inconsistencies in a specific category (numerator) by the total number of reporting forms that included the analyzed categories (denominator) (22). Variables selected for the consistency assessment were defined based on the expected logic for self-mutilation cases, where no third-party involvement occurs, information regarding the victim/perpetrator should be repeated, and data entry must be performed promptly (Table 1).

**Table 1:** Variables used to assess the consistency of self-mutilation reports among adolescents in the Notifiable Diseases Information System. Santa Catarina, 2014-2023 (n=1,403)

Variable 1	Versus	Variable 2
Number of participants (1)	vs.	Sex of the perpetrator of the violence (male or female)
Self-inflicted violence (yes)	vs.	Relationship/degree of kinship with the person assisted (own person)
Relationship/degree of kinship with the person assisted	vs.	Own person
Sex of the probable perpetrator of the violence (female)	vs.	Sex of the person assisted (female)
Gender of the person assisted (male)	vs.	Sex of the likely perpetrator of violence (male)
Life stage of the likely perpetrator of violence (2-adolescent [10-19 years])	vs.	Age (10–19 years)
Report year	vs.	Approval date
Disability/disorder (yes)	vs.	Any type of disability noted

### Data availability

Self-inflicted violence records from SINAN were requested from the Directorate of Epidemiological Surveillance, linked to the State Health Department of Santa Catarina, via email on April 19, 2024, following approval of the research project by the Research Ethics Committee of the Federal University of Santa Catarina. After review and authorization, the data were retrieved in person using a removable storage device.

### Statistical methods

To assess the quality of the self-mutilation database, the following attributes were considered: the absence of duplicate records, consistency of information, and completeness (measured by the proportion of fields filled in).

Duplicate records were evaluated in two steps, as follows.

1. Analysis of the “NDUPLIC_N” field, which identified duplicates (12): when coded as 1, the record was not a duplicate; when coded as 2, it was considered a duplicate.
2. Manual verification: names were alphabetically ordered, and the Excel function =COUNTIF(B:B;B1)>1 was applied to identify all names with more than one occurrence.

Subsequently, the mother’s name and date of occurrence were verified to confirm the absence of duplicate records. In reports of violence, including self-mutilation, careful assessment is required to determine whether it is a true duplicate record, since the same individual may be exposed to multiple episodes of violence, even on the same day, which may or may not be of the same type or nature. Therefore, all available information was thoroughly examined, with special attention to the time of occurrence and the circumstances of the event (10).

Temporal trends in completeness and consistency were analyzed using Prais–Winsten regression with Cochrane–Orcutt correction. This model is appropriate for time series with serial autocorrelation and has been applied in other studies using data from national health information systems (23). 

The dependent variables were the log-transformed proportions of completeness and consistency, as well as the number of reports. Log transformation was applied to all dependent variables to reduce the heterogeneity of the variance of the residuals. The independent variable was the year of occurrence, which allowed for the estimation of the direction and magnitude of the average annual change in the series during the study period. 

Based on the regression coefficients and standard errors, the average annual percent change (AAPC) and 95% confidence intervals (95%CI) were calculated for each dependent variable. For calculating 95%CI, the value t=2.3646 was used, considering seven degrees of freedom for the nine-year study period, according to Student’s t-test (appropriate for samples with fewer than 30 observations). Interpretation of temporal trends followed these criteria: decrease (95%CI with only negative values), increase (95%CI with only positive values), or stability (95%CI including both negative and positive values). The analyses were conducted using Stata 14.0 statistical software.

## Results 

**Figure 1:**
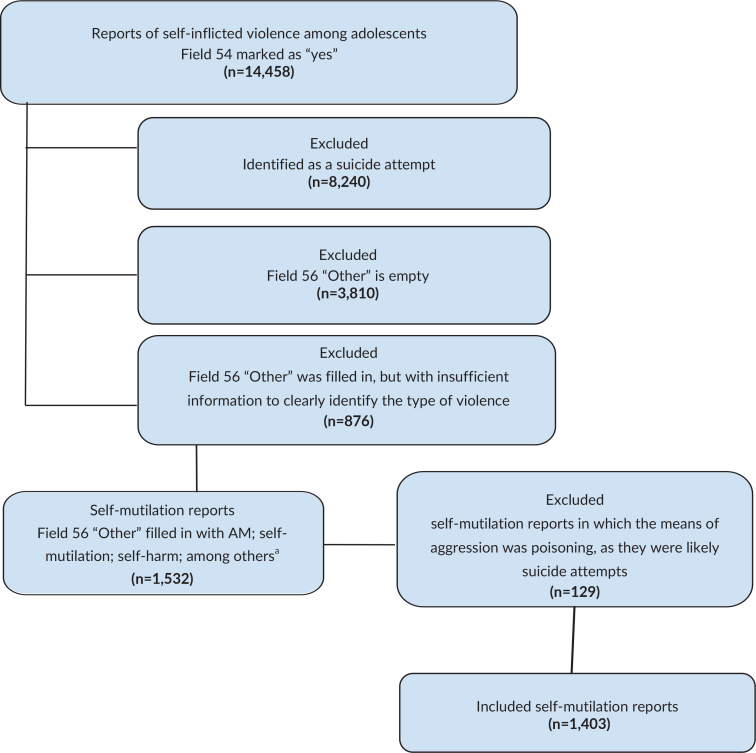
Selection of self-mutilation report records among adolescents in the Notifiable Diseases Information System. Santa Catarina, 2014-2023 (n=14,458)

^a^Fist cut; cuts; razor blade cuts; razor cuts on arms and mutilation.

All analyses were performed using Stata 14.0 statistical software. A total of 8,240 reports (57.0%) were excluded for being suicide attempts, 3,810 (26.3%) in which the “Other” field was left blank, and 876 (6.0%) in which this field was filled with insufficient information to identify the type of violence. Initially, 1,532 self-mutilation reports were identified; however, 129 (1.0%) were excluded because the means of aggression was poisoning, which was classified as a suicide attempt. Thus, 1,403 self-mutilation reports (9.7%) were selected for analysis (Figure 1). No duplicate records were identified. 

The completeness percentage was assessed for 18 variables. For 12 variables, the classification was excellent (≥95.0%). Three were classified as good—education level (90.8%), repeated violence (94.2%), and alcohol use by the perpetrator (91.2%), and two were classified as fair—sexual orientation (75.8%) and gender identity (76.2%). Completeness was very poor (46.1%) for the motivation of violence. 

The temporal trend in completeness was analyzed for 18 variables, excluding one variable (sex) that maintained 100.0% completeness throughout the period. An increasing trend was observed in three variables (race/skin color, motivation of violence, and alcohol use by the perpetrator), while the other 14 variables analyzed remained stable (Table 2).

**Table 2:** Percentage and temporal trend of completeness (C) of adolescent self-mutilation reports. Santa Catarina, 2015–2023 (n=1,403)

Check fields	2015	2016	2017	2018	2019	2020	2021	2022	2023	Total	C	Average annual change	Trend	p-value
n (%)	10 (0.7)	14 (1.0)	70 (5.0)	74 (5.3)	278 (19.8)	181 (12.9)	182 (13.0)	269 (19.2)	325 (23.2)	1,403 (100.0)				
Sex	100.0	100.0	100.0	100.0	100.0	100.0	100.0	100.0	100.0	100.0	E^a^	-	Not applicable	
Race/skin color	90.0	92.9	92.9	90.5	96.0	99.4	98.9	97.4	99.4	97.4	E	1.19 (0.32; 2.06)	Increase	0.017
Education level	100.0	100.0	90.0	77.0-	92.4	81.8	95.6	94.1	91.7	90.8	B^b^	0.22 (-3.22; 3.79)	Stability	0.885
Marital status	100.0	92.9	98.6	98.6	98.2	96.7	96.2	95.2	96.3	96.7	E	-0.08 (-0.61; 0.45)	Stability	0.724
Sexual orientation	30.0	71.4	82.9	60.8	77.0	76.8	86.3	75.8	72.0	75.8	R^c^	0.94 (-2.98; 5.03)	Stability	0.596
Gender identity	60.0	78.6	81.4	67.6	80.2	86.2	67.6	71.0	77.5	76.2	R	-0.56 (-3.32; 2.26)	Stability	0.648
Municipality of occurrence	100.0	100.0	100.0	100.0	100.0	100.0	99.5	99.6	100.0	99.9	E	-0.04 (-0.10; 0.02)	Stability	0.176
Place of occurrence	100.0	100.0	98.6	97.3	98.6	97.8	97.3	98.1	98.5	98.1	E	-0.09 (-0.51; 0.33)	Stability	0.634
Number of individuals involved	100.0	92.9	100.0	100.0	99.6	98.9	99.5	98.9	98.8	99.1	E	0.37 (-0.16; 0.90)	Stability	0.150
Motivation for the violence	20.0	21.4	34.3	25.7	37.1	47.0	44.0	56.5	55.1	46.1	MR^d^	14.15 (11.29; 17.08)	Increase	<0.001
Repeated violence	100.0	100.0	91.4	73.0	96.0	94.5	97.3	92.6	96.9	94.2	B	0.85 (-3.01; 4.88)	Stability	0.624
Alcohol use by the perpetrator	90.0	85.7	75.7	78.4	92.8	90.6	90.7	94.1	94.8	91.2	B	2.88 (0.22; 5.60)	Increase	0.042
Relationship (own person)	100.0	100.0	100.0	100.0	100.0	100.0	100.0	99.6	100.0	99.9	E	-0.02 (-0.05; 0.00)	Stability	0.060
Person sex	100.0	100.0	100.0	98.6	100.0	99.4	98.4	99.6	100.0	99.6	E	-0.05 (-0.25; 0.13)	Stability	0,512
Person’s life stage (adolescent)	100.0	92.9	98.6	100.0	100.0	98.3	97.3	99.3	100.0	99.1	E	0.45 (-0.13; 1.06)	Stability	0.120
Means of aggression: sharp object	100.0	100.0	100.0	100.0	100.0	99.4	100.0	100.0	100.0	99.9	E	-0.00 (-0.07; 0.06)	Stability	0.765
Means of aggression: blunt object	100.0	100.0	100.0	100.0	100.0	99.4	100.0	100.0	100.0	99.9	E	-0.00 (-0.07; 0.06)	Stability	0.765
Means of aggression: hot object	100.0	100.0	100.0	100.0	100.0	99.4	100.0	100.0	100.0	99.9	E	-0.00 (-0.07; 0.06)	Stability	0.765

^a^Excellent (≥95.0%); ^b^Good (90.0% -95.0%); ^c^Fair (70.0% -90.0%); ^d^Very poor (<50.0%).

Regarding consistency, six variables (85.7%) were considered excellent (≥90.0%). One variable (14.3%) was classified as fair (70.0%–89.0%), corresponding to “report date versus closure date of report.”. Temporal trends showed an increase for one variable, “sex of the person assisted (male) vs. sex of the probable perpetrator (male),” and a reduction for another, “type of disability or disorder (at least one) vs. disability or disorder (yes).” The remaining variables presented stability (Table 3).

**Table 3:** Percentage and assessment (A) of consistency of adolescent self-mutilation reporting. Santa Catarina, 2015–2023 (n=1,403)

Check fields	2015	2016	2017	2018	2019	2020	2021	2022	2023	Total	A	Average annual change	Trend	p-value
	n (%)	n (%)	n (%)	n (%)	n (%)	n (%)	n (%)	n (%)	n (%)	n (%)				
Number of individuals involved (1) vs. sex of the perpetrator of the violence (male or female)	10/10 (100.0)	13/14 (92.9)	70/70 (100.0)	74/74 (100.0)	277/278 (99.6)	179/181 (98.9)	181/182 (99.5)	266/269 (98.9)	321/325 (98.8)	1,391/1,403 (91.1)	E^a^	0.37 (-0.16; 0.90)	Stability	0.150
Self-inflicted violence (yes) vs. relationship/degree of kinship with the person assisted (own person)	10 (100.0)	13/14 (92.9)	70/70 (100.0)	74/74 (100.0)	278 (98.9)	181/181 (100.0)	181/182 (99.5)	265/269 (98.5)	322/325 (99.1)	1,391/1,403 (91.1)	E	0.38 (-0.14; 0.92)	Stability	0.136
Sex of the probable perpetrator of the violence (female) vs. sex of the person assisted (female)	8/9 (88.9)	14/14 (100.0)	50/52 (96.2)	57/59 (96.6)	221/228 (96.9)	133/136 (97.8)	145/152 (95.4)	227/230 (98.7)	265/272 (97.4)	1,120/1,152 (97.2)	E	0.10 (-0.11; 0.33)	Stability	0.298
Sex of the person assisted (male) vs. sex of the probable perpetrator of the violence (male)	1/1 (100.0)	-	18/20 (90.0)	15/16 (93.8)	50/57 (87.7)	45/46 (97.8)	30/34 (88.2)	39/41 (95.1)	53/60 (88.3)	251/275 (91.3)	E	40.68 (4.17; 89.99)	Increase	0.036
Life stage of the probable perpetrator of the violence (adolescent) vs. age (10–19 years)	9/10 (90.0)	12/14 (85.7)	69/70 (98.6)	71/74 (95.9)	254/278 (91.4)	164/181 (90.6)	167/182 (91.8)	251/269 (93.3)	311/325 (97.7)	1,308/1,403 (93.2)	E	0.57 (-1.00; 2.18)	Stability	0.424
Report date vs. closure date (must be equal)	8/10 (80.0)	12/14 (85.7)	61/70 (87.1)	43/74 (58.1)	209/278 (75.1)	151/181 (83.4)	128/182 (70.3)	178/269 (65.4)	259/325 (79.6)	147/1403 (74.6)	R^b^	-1.74 (-5.59; 2.25)	Stability	0.336
Type of disability or disorder (at least one) vs. disability or disorder (yes)	5/5 (100.0)	7/7 (100.0)	35/36 (97.2)	22/22 (100.0)	69/72 (95.8)	45/47 (95.7)	37/39 (95.7)	67/71 (94.4)	121/123 (98.4)	408/422 (96.7)	E	-0.62 (-0.98; -0.25)	Decrease	0.007

^a^Excellent (≥90.0%); ^b^Fair (70.0%-89.0%).

## Discussion 

Among the reports of self-inflicted violence, one in ten was identified as self-mutilation; however, it was not possible to obtain the type of violence in just over 30.0% of the reports, suggesting that this number may be underestimated. There was no record of duplication, indicating that the procedures guided by the Ministry of Health are being implemented at the municipal level (12). Completeness and consistency were excellent for most variables. 

In the analysis of completeness, the classifications good and excellent predominated. However, they were still lower than in two studies evaluating the quality of SINAN records in Santa Catarina on child sexual violence, with 93.2% (14), and sexual violence against women, with 93.3% (13). Consistency was excellent in most records, slightly lower than in the comparative studies, with 90.0% (14) and 98.9% (13), respectively. A relevant characteristic among the studies refers to the difference in recording the type of violence: in cases of sexual violence, there is a specific closed field for its identification, whereas in cases of self-mutilation, the entry occurs in an open field and requires manual description of the type of violence. It is considered essential to insert a closed field for recording the type of self-inflicted violence, with options for suicide attempt and self-mutilation, in a future update of the reporting form.

Among the variables assessed as excellent, half were mandatory fields, and the other half were key fields. Although the SINAN guidelines stipulate that failure to complete mandatory fields renders it impossible to include the report in the system (10), the option “unknown” is still accepted as a valid response. As a result, the report is recorded, but without providing effective information about the event, which compromises data quality and maintains levels of incompleteness even in fields considered mandatory. In this context, it is suggested that the rules for filling out the system be revised to expand the mandatory fields that require valid responses, while restricting the use of the “Unknown” option. 

Repeated self-mutilation has been evaluated with good completeness and stability in the temporal trend. This is a relevant aspect. In Sweden, between 2018 and 2019, 53,172 people were treated in hospitals after self-mutilation; this indicated a prevalence of repeated self-mutilation of 4.0% after one month and 9.0% after six months, which highlighted the need for follow-up strategies and early intervention (24). Recognition of recurrent episodes of self-mutilation and proper completion of the form may contribute to prevention and to avoiding the worsening of cases.

The suspected use of alcohol was evaluated with good completeness and an increasing temporal trend. The National Survey of School Health (Pesquisa Nacional de Saúde Escolar, PeNSE), conducted in 2019, concluded that earlier exposure to alcohol is a factor that may have health consequences. The Survey found that 34.6% of adolescents aged 13–17 years had first consumed alcoholic beverages before the age of 14 (25). A scoping review on self-mutilation, suicide, and alcohol consumption in Sri Lanka concluded that alcohol use is an important risk factor in cases of self-mutilation. It emphasized that targeted interventions addressing problematic use are necessary to prevent this type of violence (26). To improve the quality of this information, it is essential to strengthen the process of completing this field in the reporting form through continuing education of health professionals.

Sexual orientation and gender identity showed fair completeness with a stable temporal trend. The age group selected for this study may have influenced these results, as although these are self-reported and mandatory fields for individuals over 10 years old, special care is required when dealing with children and adolescents, as they are still developing these characteristics (10). On the other hand, it is necessary to raise awareness among professionals about the proper reporting of violence against the lesbian, gay, bisexual, and transgender (LGBT) population. Discriminatory and exclusionary care, still present in many contexts, represents a significant barrier to access to health services for this population, particularly for transgender individuals (27).

The completeness of the variable referring to the motivation for violence was classified as very poor, but with an increasing temporal trend. This field is still little explored in studies; however, in the LGBT population in the state of Rio de Janeiro, similar findings were observed, indicating that in most reports, the reason for the violence was not recorded (73.9%) during the period 2015–2021 (27). This highlights that completing this variable is a challenging task. Although it is a mandatory field, it still presents gaps that could be minimized through continuing education for professionals to ensure the proper completion of reporting forms. Although the completion manual is available, some fields in the form involve concepts that have only recently been incorporated into society, which may still be difficult for professionals to understand, resulting in completion errors (28). 

Most variables were assessed as excellent in terms of consistency. The report date vs. the closure date was fair. Accurate completion of these fields is important to verify whether the intervention was carried out promptly (10). Since 2015, all cases of violence have been closed at the time of reporting (10). The regular consistency of this variable could be improved, given that reporting forms should be reviewed, whenever possible, by the Epidemiological Surveillance Centers or at the municipal level before being submitted for inclusion in the system (12). One possible solution would be to implement automatic completion of this field or to block form saving if the dates do not match, thereby ensuring the accuracy and integrity of the recorded data.

Among the main limitations of this study, it is acknowledged that including a larger number of time points could have increased the robustness of the analyses. However, with the new version of the reporting form in 2014 (20), it was possible to include data from previous years. The final year, 2023, was adopted as it was the most recent period with consolidated data available at the time of the research. It is recommended that future studies explore longer time series. The geographic limitation is also considered a constraint; therefore, it is suggested that this study be replicated in other states or at the national level, providing a broader view of the quality of self-mutilation reports among adolescents in SINAN. 

Another important limitation is the absence or inadequate completion of the open field corresponding to the type of self-inflicted violence, accounting for just over 30.0%, resulting in a high number of excluded reports. Although this study presents a good assessment of the analyzed reports, it is important to consider that the results could vary if information on the type of self-inflicted violence were available in the excluded reports. Although there are some limitations, the findings should not be invalidated, as they are relevant to the understanding and monitoring of self-mutilation.

To improve the quality of self-mutilation data in SINAN, it is recommended that future updates of the reporting form include a closed field with options for suicide attempt and self-mutilation, as well as an expansion of the number of mandatory fields. Continuing education of health professionals is also recommended, especially those working in services that attend adolescents, to ensure proper identification, reporting, and referral, as well as constant monitoring and analysis of reports by the responsible agencies.

The results of this study indicate that most variables of self-mutilation reports among adolescents in SINAN in Santa Catarina exhibit good to excellent levels of completeness and consistency. No duplicate records were identified. The analysis of temporal trends revealed stability in the completeness and consistency of information during the study period. 
