# Supplementary material for: Completeness, consistency, and duplicity of self-mutilation reports by adolescents in the Notifiable Diseases Information System: evaluation study, Santa Catarina, 2014-2023
Source: Epidemiol Serv Saude. 2025 Nov 28;35:e20250412. doi: 10.1590/S2237-96222026v35e20250412.en (PMC12662402; doi:10.1590/S2237-96222026v35e20250412.en)
Supplement: Translation

# Translation: Completitude, consistência e duplicidade das notificações de automutilação por adolescentes no Sistema de Informação de Agravos de Notificação: estudo de avaliação, Santa Catarina,

**DOI:** 10.1590/S2237-96222026v35e20250412.pt

**Authors:** Thayse de Paula Pinheiro, Deise Warmling, Doroteia Aparecida Hofelmann, Elza Berger Salema Coelho

**Affiliations:** 1 Universidade Federal de Santa Catarina, Faculdade de Saúde Pública, Florianópolis, SC, Brasil; 2 Universidade Federal do Paraná, Curitiba, PR, Brasil

**Keywords:** Automutilação, Adolescentes, Notificação, Sistema de Informação de Agravos de Notificação, Estudo de Avaliação

## Abstract

**Objetivo:**

Avaliar a completitude, consistência e duplicidade das notificações de automutilação por adolescentes, em Santa Catarina, de 2014 a 2023.

**Métodos:**

Tratou-se de estudo de avaliação das notificações do Sistema de Informação de Agravo de Notificação (Sinan). Analisou-se a completitude de 18 variáveis e classificou-se como excelente (≥95,0%), bom (90,0%-95,0%), regular (70,0%-90,0%), ruim (50,0%-70,0%) e muito ruim (<50,0%). A consistência de sete variáveis foi analisada e classificada como: excelente (≥90,0%), regular (70,0%-89,0%) e baixa consistência (<70,0%). Checou-se a duplicidade a partir da variável “NDUPLIC_N” e por verificação manual. Analisou-se a tendência temporal pela regressão de Prais-Winsten, classificando em estabilidade, aumento e redução.

**Resultados:**

Foram identificadas 1.403 notificações de automutilação. A completitude foi excelente em 12 variáveis e boa em três. Orientação sexual (75,8%) e identidade de gênero (76,2%) foram avaliadas como regulares, e motivação da violência (46,1%) foi avaliada como muito ruim. Três variáveis mostraram tendência de aumento de completitude. A consistência foi excelente em seis variáveis e regular em uma, com tendência de aumento no registro de “sexo da pessoa atendida versus sexo do autor”, redução em “presença de deficiência ou transtorno vs. tipo” e estabilidade nas demais. Não houve duplicidades.

**Conclusão:**

Os dados analisados mostraram boa qualidade no registro de automutilação por adolescentes no Sinan de Santa Catarina. Estratégias como educação continuada dos profissionais de saúde e alterações na ficha de notificação são essenciais para a qualificação dos registros e o planejamento de ações para o enfrentamento dessa violência.

**Aspectos éticos**

Esta pesquisa respeitou os princípios éticos, obtendo os seguintes dados de aprovação:

Comitê de ética em pesquisa: Universidade Federal de Santa Catarina

Número do parecer: 6.775.433

Data de aprovação: 19/4/2024

Certificado de apresentação de apreciação ética: 76226423.8.0000.0121

Registro do consentimento livre e esclarecido: Dispensado.

## Introdução 

A automutilação é caracterizada por danos autoinfligidos sem o intuito de tirar a própria vida (1). A gravidade pode variar, de acordo com a frequência e intensidade das lesões, sendo classificada em leve, moderada e severa (1). A prática da automutilação, geralmente, começa na adolescência e é considerada um fator que impacta negativamente o desenvolvimento dessa etapa de transição da infância para a vida adulta, caracterizada por transformações corporais, cognitivas, afetivas, sociais e comportamentais (2). 

Identificou-se a prevalência de automutilação de 17,6% entre adolescentes de 17 países localizados na América do Norte, Ásia, Europa e Oceania (3). Na Noruega, entre 2017 e 2018, em uma amostra de 37.268 adolescentes, essa taxa foi 16,1% (4). Na Tailândia, em 2020, entre 1.457 jovens, 5,4% haviam se automutilado (5). Em 2021, na China, entre 1.180 estudantes, observou-se a prevalência de 24,0% (6). A variação na prevalência global de automutilação pode ser atribuída a fatores diversos, como aspectos socioculturais (relações familiares e sociais; e região domiciliar) ou metodológicos (tipo de amostragem, comunitária ou clínica; recorte temporal; e sexo dos participantes) (3).

No Brasil, a ocorrência de automutilação também apresenta variações. Em 2018, entre 517 estudantes mineiros de 10-14 anos, a proporção foi de 9,5% de automutilação, com cinco ou mais ocorrências em 2016 (7). Em 2017, em domicílios de 505 adolescentes maceioenses de 12-17 anos, identificou-se prevalência ligeiramente inferior, 6,5% (8). Por outro lado, em 2019, observou-se prevalência consideravelmente mais elevada em investigação realizada com 878 estudantes de municípios do interior do Rio Grande do Sul, onde 53,0% dos participantes relataram automutilação (9). A pesquisa destacou fatores que podem ter influenciado nesses resultados como: o contágio social, o estigma e a falta de privacidade em comunidades menores, o acesso à internet e às redes sociais e a escassez de atividades (9).

A prevalência e as características da automutilação podem ser verificadas no Sistema de Informação de Agravos de Notificação (Sinan). Em 2014, o instrumento de notificação foi modificado, passando a se chamar Ficha de Notificação/Investigação de Violência Interpessoal/Autoprovocada, o que aumentou a atenção às violências autoprovocadas (10). A tentativa de suicídio passou a ser de notificação imediata (em até 24 horas) na esfera municipal, com o objetivo de garantir a intervenção adequada aos casos (10). A automutilação deve ser notificada por todos os profissionais de saúde, preferencialmente por aquele que realizou o atendimento (11), sendo relevante para a identificação, o encaminhamento e a prevenção dos casos de automutilação.

Para fortalecer o sistema de vigilância desse agravo, é fundamental uma base de dados de boa qualidade com informações completas, fidedignas aos dados originais, sem duplicidades e com os campos preenchidos de forma consistente (12). Pesquisas nacionais semelhantes foram encontradas em Santa Catarina (13,14), mas com foco na análise do preenchimento das notificações de violência sexual. Em Niterói, foi analisado o grau de completitude nas notificações de violência contra pessoa idosa (15), e, em Mato Grosso do Sul, analisaram-se a completitude e a consistência com foco nas mulheres indígenas (16). 

O Sinan é a principal ferramenta de registro de automutilação, no âmbito da saúde, no Brasil (10). Até o momento desta pesquisa, não foi encontrado nenhum estudo brasileiro que abordasse a qualidade dos registros de automutilação, tornando esta produção relevante e inovadora. Os resultados poderão subsidiar ações nas três esferas de governo, visando à melhoria da qualidade dos dados relacionados a esse tipo de violência autoprovocada. 

Este estudo teve como objetivo avaliar a completitude, a consistência e a não duplicidade nas notificações de automutilação por adolescentes, no Sistema de Informação de Agravos de Notificação, em Santa Catarina, de 2014 a 2023.

## Métodos 

### Delineamento do estudo e contexto

Tratou-se de estudo de avaliação, do tipo normativa, sobre a qualidade das notificações de automutilação em adolescentes registradas no Sinan (17). Santa Catarina está localizada na região Sul do Brasil, com população estimada em 8.058.441 habitantes em 2024 (18). Segundo as projeções, em 2025, a população de adolescentes no estado é de 1.032.531, sendo 531.693 do sexo masculino e 500.838 do feminino (19).

O ano inicial do estudo foi 2014, quando ocorreu a mudança no instrumento de coleta de dados, que passou a chamar-se Ficha de Notificação/Investigação de Violência Interpessoal/Autoprovocada(20). Nessa atualização da ficha, o instrutivo de preenchimento orienta claramente sobre a necessidade de especificar o tipo de violência autoprovocada (10). O ano final do estudo, 2023, foi determinado pela disponibilidade dos dados no momento da realização da pesquisa.

### Participantes 

Foram incluídas as notificações de automutilação, entre os registros de violência autoprovocada, por adolescentes (10-19 anos). A definição de automutilação adotada segue a que foi estabelecida pelo Ministério da Saúde, sendo caracterizada como a ação em que a pessoa provoca agressão contra si mesma, geralmente com cortes e queimaduras, sem intencionalidade suicida (21). A faixa etária adotada na Vigilância de Violências e Acidentes é convergente com a Organização Mundial da Saúde (10). 

Para identificar as notificações de automutilação, Conforme o instrutivo de preenchimento do Sinan, ao preencher o campo 54 com “1 – Sim” para violência autoprovocada, o campo 56 devia ser preenchido com “1 – Sim” no quadrículo “Outros”. Devia também ser descrita a violência autoprovocada (se referiu à automutilação ou à tentativa de suicídio) (10). Tratou-se de um campo aberto no qual era necessário escrever por extenso (21). Foram incluídas as notificações com o campo “outros” preenchido como automutilação. Os registros em branco, referentes à tentativa de suicídio ou insuficientes para identificar o tipo de violência autoprovocada, foram excluídos. Essa estratégia teve como objetivo minimizar o viés de seleção, assegurando a consistência dos casos analisados.

### Variáveis 

A qualidade dos registros referiu-se à duplicidade, completitude e consistência. A variável da ficha de notificação “NDUPLIC_N” foi observada para a análise da duplicidade.

Em relação à completitude, foram avaliadas as seguintes variáveis de preenchimento obrigatório: sexo; orientação sexual; identidade de gênero; município de ocorrência; local de ocorrência; motivação da violência; vínculo/grau de parentesco com a pessoa atendida; sexo do provável autor da violência; ciclo de vida do provável autor da violência (10-19 anos) e de preenchimento essencial: raça/cor da pele; escolaridade; situação conjugal/estado civil; se ocorreu outras vezes (violência de repetição); suspeita de uso de álcool; número de envolvidos e três meios da agressão (objeto perfurocortante; objeto contundente e substância/objeto quente) (12). O não preenchimento do campo obrigatório impossibilitou a inclusão da notificação no Sinan. O campo essencial foi aquele que registrou dado necessário à investigação do caso ou ao cálculo de indicador epidemiológico ou operacional (10). 

A completitude dos registros referiu-se ao preenchimento dos campos na ficha de notificação e cada um deles teve finalidade específica, que visou à caracterização da violência, à análise da situação e à implementação de medidas de prevenção (12). A completitude foi mensurada com base na proporção de registros que apresentaram campos ignorados ou em branco, em cinco categorias: excelente (≥95,0%), bom (90,0%-95,0%), regular (70,0%-90,0%), ruim (50,0%-70,0%) e muito ruim (<50,0%) (12). Foram considerados campos incompletos os ignorados (nº 9) e os vazios (missing). 

A avaliação da consistência teve o objetivo de detectar dados preenchidos incorretamente nas fichas de notificação e a posterior correção (12). A consistência foi determinada pela proporção de variáveis relacionadas que possuíam valores coerentes e sem contradições e classificada em diferentes níveis: excelente (com níveis de coerência iguais ou superiores a 90,0%), regular (70,0%-89,0%) e baixa consistência (abaixo de 70,0%). 

O percentual de inconsistência foi calculado por meio da divisão do número de fichas de notificação com inconsistências em uma categoria específica (numerador) pelo total de fichas de notificação que incluíam as categorias analisadas (denominador) (22). As variáveis selecionadas para a avaliação da consistência foram definidas com base na lógica esperada para os casos de automutilação, onde não houve a participação de terceiros, as informações sobre a vítima/autor devem ser repetidas e a realização deve ser feita em tempo oportuno (Tabela 1).

**Tabela 1:** Variáveis utilizadas para a verificação de consistência das notificações de automutilação por adolescentes no Sistema de Informação de Agravos de Notificação. Santa Catarina, 2014-2023 (n=1.403)

Variável 1	Versus	Variável 2
Número de envolvidos (1)	vs.	Sexo do autor da agressão (masculino ou feminino)
Violência autoprovocada (sim)	vs.	Vínculo/grau de parentesco com a pessoa atendida (própria pessoa)
Vínculo/grau de parentesco com a pessoa atendida	vs.	Própria pessoa
Sexo do provável autor da violência (feminino)	vs.	Sexo da pessoa atendida (feminino)
Sexo da pessoa atendida (masculino)	vs.	Sexo do provável autor da violência (masculino)
Ciclo de vida do provável autor da violência (2 – adolescente [10-19 anos])	vs.	Idade (10-19 anos)
Data da notificação	vs.	Data de encerramento
Deficiência/transtorno (sim)	vs.	Qualquer tipo de deficiência assinalada

### Fonte de dados

Os registros de violência autoprovocada do Sinan foram solicitados à Diretoria de Vigilância Epidemiológica, vinculada à Secretaria de Estado da Saúde de Santa Catarina, por e-mail em 19 de abril de 2024, após a aprovação do projeto de pesquisa no Comitê de Ética em Pesquisa da Universidade Federal de Santa Catarina. Posteriormente à análise e à autorização, os dados foram retirados pessoalmente por meio de dispositivo de armazenamento removível.

### Métodos estatísticos

Para verificar a qualidade da base de dados de automutilação, foram considerados os seguintes atributos: ausência de duplicidades, consistência (das informações) e completitude (proporção de campos preenchidos) dos registros.

A duplicidade dos registros foi avaliada em duas etapas, conforme a seguir.

1. Análise do campo “NDUPLIC_N”, que caracterizou duplicidades (12): quando preenchido com o número 1, não era duplicidade; quando preenchido com o número 2, era duplicidade.
2. Verificação manual: ordenaram-se os nomes por ordem alfabética e aplicou-se a função =COUNTIF(B:B;B1)>1 no Excel para a identificação de todos os nomes com mais de uma ocorrência.

Em seguida, foi verificado se o nome da mãe e a data de ocorrência eram iguais para caracterização da duplicidade. Nas notificações de violência, incluindo as de automutilação, é importante avaliar cautelosamente quando se trata de uma duplicidade verdadeira, pois uma pessoa pode ser exposta a vários episódios de violência, inclusive no mesmo dia, podendo ser ou não do mesmo tipo ou natureza. Portanto, todas as informações foram devidamente observadas, considerando especialmente o horário da ocorrência e as circunstâncias do evento (10).

Foram analisadas as tendências temporais de completitude e consistência a partir da regressão de Prais-Winsten com correção de Cochrane-Orcutt. Os modelos foram adequados para séries temporais com autocorrelação serial e utilizadas por outros estudos, com dados oriundos de sistemas nacionais de informação em saúde (23). 

As variáveis dependentes foram as proporções logaritimizadas de completitude e consistência, além do número de notificações. Aplicou-se a transformação logarítmica em todas as variáveis dependentes a fim de reduzir a heterogeneidade da variância dos resíduos. A variável independente foi o ano de ocorrência, permitindo estimar a direção e a magnitude da variação anual média da série no período analisado. 

A partir dos coeficientes e erros-padrão obtidos nas análises de regressão, calcularam-se a variação percentual média anual e os intervalos de confiança de 95% (IC95%) para cada variável dependente analisada. No cálculo dos IC95%, utilizou-se o valor t=2,3646, considerando 7 graus de liberdade no período avaliado de nove anos, referente ao teste t de Student (adequado para amostras com menos de 30 observações). A interpretação da tendência temporal seguiu os critérios: redução (IC95% com valores negativos), aumento (IC95% com valores positivos) ou estabilidade (IC95% incluindo valores negativos e positivos). As análises foram conduzidas no software estatístico Stata 14.0.

## Resultados 

Ocorreram 14.458 notificações de violência autoprovocada por adolescentes no período analisado. Foram excluídas 8.240 (57,0%) por serem tentativas de suicídio, 3.810 (26,3%) em que o campo “outros” estava vazio e 876 (6,0%) nas quais o campo “outros” foi preenchido com informações insuficientes para o reconhecimento do tipo de violência. Inicialmente, identificaram-se 1.532 notificações de automutilação, contudo foram excluídos 129 (1,0%), pois o meio da agressão foi envenenamento, relativo à tentativa de suicídio. Foram selecionadas 1.403 notificações de automutilação (9,7%) para a análise (Figura 1). Não houve duplicidades. 

**Figura 1:** Seleção de registros de notificação de automutilação em adolescentes no Sistema de Informação de Agravos de Notificação. Santa Catarina, 2014-2023 (n=14.458)

^a^Corte de punho; cortes; cortes com lâmina de barbear; cortes de navalho nos braços e mutilação.

O percentual de completitude foi analisado a partir de 18 variáveis. Em 12 variáveis, a classificação foi excelente (≥95,0%). Três classificaram-se como boas – escolaridade (90,8%), violência de repetição (94,2%) e uso de álcool agressor (91,2%) – e duas, como regulares – orientação sexual (75,8%) e identidade de gênero (76,2%). A completitude foi muito ruim (46,1%) para motivação da violência. 

A tendência temporal da completitude foi analisada em 18 variáveis, sendo uma excluída (sexo), pois manteve 100,0% de completitude no período. Observaram-se tendência de aumento em três (raça/cor da pele, motivação da violência e uso de álcool agressor) e estabilidade nas outras 14 variáveis analisadas (Tabela 2).

**Tabela 2:** Percentual e tendência temporal da completitude (C) das notificações de automutilação por adolescentes. Santa Catarina, 2015-2023 (n=1.403)

Campos de checagem	2015	2016	2017	2018	2019	2020	2021	2022	2023	Total	C	Variação média anual	Tendência	p-valor
n (%)	10 (0,7)	14 (1,0)	70 (5,0)	74 (5,3)	278 (19,8)	181 (12,9)	182 (13,0)	269 (19,2)	325 (23,2)	1.403 (100,0)				
Sexo	100,0	100,0	100,0	100,0	100,0	100,0	100,0	100,0	100,0	100,0	E^a^	-	Não se aplica	
Raça/cor da pele	90,0	92,9	92,9	90,5	96,0	99,4	98,9	97,4	99,4	97,4	E	1,19 (0,32; 2,06)	Aumento	0,017
Escolaridade	100,0	100,0	90,0	77,0-	92,4	81,8	95,6	94,1	91,7	90,8	B^b^	0,22 (-3,22; 3,79)	Estabilidade	0,885
Situação conjugal	100,0	92,9	98,6	98,6	98,2	96,7	96,2	95,2	96,3	96,7	E	-0,08 (-0,61; 0,45)	Estabilidade	0,724
Orientação sexual	30,0	71,4	82,9	60,8	77,0	76,8	86,3	75,8	72,0	75,8	R^c^	0,94 (-2,98; 5,03)	Estabilidade	0,596
Identidade de gênero	60,0	78,6	81,4	67,6	80,2	86,2	67,6	71,0	77,5	76,2	R	-0,56 (-3,32; 2,26)	Estabilidade	0,648
Município de ocorrência	100,0	100,0	100,0	100,0	100,0	100,0	99,5	99,6	100,0	99,9	E	-0,04 (-0,10; 0,02)	Estabilidade	0,176
Local da ocorrência	100,0	100,0	98,6	97,3	98,6	97,8	97,3	98,1	98,5	98,1	E	-0,09 (-0,51; 0,33)	Estabilidade	0,634
Número de envolvidos	100,0	92,9	100,0	100,0	99,6	98,9	99,5	98,9	98,8	99,1	E	0,37 (-0,16; 0,90)	Estabilidade	0,150
Motivação da violência	20,0	21,4	34,3	25,7	37,1	47,0	44,0	56,5	55,1	46,1	MR^d^	14,15 (11,29; 17,08)	Aumento	<0,001
Violência de repetição	100,0	100,0	91,4	73,0	96,0	94,5	97,3	92,6	96,9	94,2	B	0,85 (-3,01; 4,88)	Estabilidade	0,624
Uso de álcool agressor	90,0	85,7	75,7	78,4	92,8	90,6	90,7	94,1	94,8	91,2	B	2,88 (0,22; 5,60)	Aumento	0,042
Vínculo (própria pessoa)	100,0	100,0	100,0	100,0	100,0	100,0	100,0	99,6	100,0	99,9	E	-0,02 (-0,05; 0,00)	Estabilidade	0,060
Sexo do autor	100,0	100,0	100,0	98,6	100,0	99,4	98,4	99,6	100,0	99,6	E	-0,05 (-0,25; 0,13)	Estabilidade	0,512
Ciclo de vida do autor (adolescente)	100,0	92,9	98,6	100,0	100,0	98,3	97,3	99,3	100,0	99,1	E	0,45 (-0,13; 1,06)	Estabilidade	0,120
Meio da agressão perfurocortante	100,0	100,0	100,0	100,0	100,0	99,4	100,0	100,0	100,0	99,9	E	-0,00 (-0,07; 0,06)	Estabilidade	0,765
Meio da agressão objeto contundente	100,0	100,0	100,0	100,0	100,0	99,4	100,0	100,0	100,0	99,9	E	-0,00 (-0,07; 0,06)	Estabilidade	0,765
Meio da agressão objeto quente	100,0	100,0	100,0	100,0	100,0	99,4	100,0	100,0	100,0	99,9	E	-0,00 (-0,07; 0,06)	Estabilidade	0,765

^a^Excelente (≥95,0%); ^b^Bom (90,0%-95,0%); ^c^Regular (70,0%-90,0%); ^d^Muito ruim (<50,0%).

No que se refere à consistência, seis variáveis (85,7%) foram consideradas excelentes (≥90,0%). Houve uma variável (14,3%) de consistência regular (70,0%-89,0%), correspondente à “data de notificação versus a data de encerramento da notificação”. Verificaram-se tendências temporais de aumento para uma variável, “sexo da pessoa atendida (masculino) vs. sexo do provável autor da violência” (masculino), e de redução também para uma, “tipo de deficiência ou transtorno (ao menos um) vs. deficiência ou transtorno (sim)”. As demais apresentaram estabilidade (Tabela 3).

**Tabela 3:** Percentual de consistência e avaliação (A) das notificações de automutilação por adolescentes. Santa Catarina, 2015-2023 (n=1.403)

Campos de checagem	2015	2016	2017	2018	2019	2020	2021	2022	2023	Total	A	**Variação m**édia anual	Tendência	p-valor
	n (%)	n (%)	n (%)	n (%)	n (%)	n (%)	n (%)	n (%)	n (%)	n (%)				
Número de envolvidos (1) vs. sexo do autor da agressão (masculino ou feminino)	10/10 (100,0)	13/14 (92,9)	70/70 (100,0)	74/74 (100,0)	277/278 (99,6)	179/181 (98,9)	181/182 (99,5)	266/269 (98,9)	321/325 (98,8)	1.391/1403 (91,1)	E^a^	0,37 (-0,16; 0,90)	Estabilidade	0,150
Violência autoprovocada (sim) vs. vínculo/grau de parentesco com a pessoa atendida (própria pessoa)	10 (100,0)	13/14 (92,9)	70/70 (100,0)	74/74 (100,0)	275/278 (98,9)	181/181 (100,0)	181/182 (99,5)	265/269 (98,5)	322/325 (99,1)	1.391/1403 (91,1)	E	0,38 (-0,14; 0,92)	Estabilidade	0,136
Sexo do provável autor da violência (feminino) vs. sexo da pessoa atendida (feminino)	8/9 (88,9)	14/14 (100,0)	50/52 (96,2)	57/59 (96,6)	221/228 (96,9)	133/136 (97,8)	145/152 (95,4)	227/230 (98,7)	265/272 (97,4)	1.120/1152 (97,2)	E	0,10 (-0,11; 0,33)	Estabilidade	0,298
Sexo da pessoa atendida (masculino) vs. sexo do provável autor da violência (masculino)	1/1 (100,0)	-	18/20 (90,0)	15/16 (93,8)	50/57 (87,7)	45/46 (97,8)	30/34 (88,2)	39/41 (95,1)	53/60 (88,3)	251/275 (91,3)	E	40,68 (4,17; 89,99)	Aumento	0,036
Ciclo de vida do provável autor da violência (adolescente) vs. idade (10-19 anos)	9/10 (90,0)	12/14 (85,7)	69/70 (98,6)	71/74 (95,9)	254/278 (91,4)	164/181 (90,6)	167/182 (91,8)	251/269 (93,3)	311/325 (97,7)	1.308/1403 (93,2)	E	0,57 (-1,00; 2,18)	Estabilidade	0,424
Data de notificação vs. data de encerramento (devem ser iguais)	8/10 (80,0)	12/14 (85,7)	61/70 (87.1)	43/74 (58,1)	209/278 (75,1)	151/181 (83,4)	128/182 (70,3)	178/269 (65,4)	259/325 (79,6)	147/1403 (74,6)	R^b^	-1,74 (-5,59; 2,25)	Estabilidade	0,336
Tipo de deficiência ou transtorno (ao menos um) vs. deficiência ou transtorno (sim)	5/5 (100,0)	7/7 (100,0)	35/36 (97,2)	22/22 (100,0)	69/72 (95,8)	45/47 (95,7)	37/39 (95,7)	67/71 (94,4)	121/123 (98,4)	408/422 (96,7)	E	-0,62 (-0,98; -0,25)	Redução	0,007

^a^Excelente (≥90,0%); ^b^Regular (70,0%-89,0%).

## Discussão 

Entre as notificações de violência autoprovocada, uma em cada dez foi identificada como automutilação, entretanto não foi possível obter o tipo de violência em pouco mais de 30,0% das notificações, sugerindo que esse número possa estar subestimado. Não houve registro de duplicidades, o que demonstra que os procedimentos orientados pelo Ministério da Saúde estão sendo efetuados em nível municipal (12). A completitude e a consistência foram excelentes na maior parte das variáveis. 

Na análise de completitude, as classificações boa e excelente predominaram, porém ainda inferior a dois estudos de avaliação da qualidade dos registros do Sinan de Santa Catarina sobre violência sexual infantil, com 93,2% (14), e violência sexual contra a mulher, com 93,3% (13). A consistência se mostrou excelente na maioria dos registros, ligeiramente menor que nos estudos comparados, com 90,0% (14) e 98,9% (13), respectivamente. Uma característica relevante entre os estudos refere-se à diferença no registro do tipo de violência: nos casos de violência sexual, há um campo fechado específico para sua identificação, enquanto, nos casos de automutilação, o preenchimento ocorre em campo aberto e exige a descrição manual do tipo de violência. Considera-se fundamental a inserção de campo fechado para o registro do tipo de violências autoprovocadas, com opção para tentativa de suicídio e automutilação, em atualização futura da ficha de notificação.

Entre as variáveis avaliadas como excelente, metade era de preenchimento obrigatório, e a outra metade, de preenchimento essencial. Embora o instrutivo do Sinan estabeleça que o não preenchimento de campos obrigatórios inviabiliza a inclusão da notificação no sistema (10), a opção “ignorado” ainda é aceita como resposta válida. Com isso, a notificação é registrada, porém sem fornecer a informação efetiva sobre o agravo, o que compromete a qualidade do dado e mantém níveis de incompletitude mesmo em campos considerados obrigatórios. Diante desse cenário, sugere-se a revisão das regras de preenchimento do sistema, com a ampliação dos campos obrigatórios que exijam respostas válidas, restringindo o uso da opção “ignorado”.

A automutilação de repetição foi avaliada com boa completitude e estabilidade na tendência temporal. Trata-se de aspecto relevante. Na Suécia, entre 2018 e 2019, 53.172 pessoas foram atendidas em hospitais após a prática de automutilação; isso indicou a prevalência de repetição da automutilação de 4,0% após um mês e de 9,0% após seis meses, o que evidenciou a necessidade de estratégias de acompanhamento e intervenção precoce (24). O reconhecimento dos episódios recorrentes de automutilação e o preenchimento correto da ficha podem favorecer a prevenção e o agravamento dos casos.

A suspeita de uso de álcool foi avaliada com boa completitude e aumento na tendência temporal. A Pesquisa Nacional de Saúde do Escolar, em 2019, concluiu que a exposição mais precoce ao álcool é um fator que pode acarretar consequências à saúde. A Pesquisa apurou que 34,6% dos adolescentes de 13-17 anos havia ingerido bebida alcoólica pela primeira vez com menos de 14 anos (25). Em revisão de escopo sobre automutilação, suicídio e consumo de álcool, no Sri Lanka, concluiu-se que o consumo de álcool é um fator de risco importante nos casos de automutilação e afirmou que intervenções direcionadas ao uso problemático são necessárias para prevenir esse tipo de violência (26). Para aprimorar o conhecimento dessas informações, torna-se indispensável a qualificação, por meio de educação continuada dos profissionais de saúde, do processo de preenchimento desse dado na ficha de notificação.

A orientação sexual e a identidade de gênero apresentaram completitude regular com tendência de estabilidade. A faixa etária selecionada para este estudo pode ter influenciado nesses resultados, pois, mesmo sendo um campo autodeclaratório e obrigatório para maiores de 10 anos, exige-se cuidado especial quando se trata de criança ou adolescente por estarem em fase de desenvolvimento dessas características (10). Por outro lado, faz-se necessário sensibilizar os profissionais para o registro adequado da notificação de violência contra a população lésbica, gay, bissexual e transgênero (LGBT). O atendimento discriminatório e excludente, ainda presente em muitos contextos, configura-se como barreira significativa ao acesso dessa população aos serviços de saúde, especialmente no caso de pessoas transgêneras (27).

A completitude da variável referente à motivação para a violência foi avaliada como muito ruim, porém com tendência temporal de aumento. Esse é um campo ainda pouco explorado em estudos; entretanto, na população LGBT no estado do Rio de Janeiro, houve resultados semelhantes, os quais indicaram que, na maioria das notificações, não foi registrado o motivo da violência (73,9%) no período 2015-2021 (27). Isso sinaliza que o preenchimento dessa variável é desafiador. Apesar de ser campo obrigatório, observa-se que ainda apresenta lacunas que poderiam ser minimizadas com a educação continuada dos profissionais para o preenchimento adequado das fichas de notificação. Embora o instrutivo de preenchimento esteja disponível, alguns campos da ficha contemplam conceitos incorporados recentemente na sociedade que ainda podem ser de difícil compreensão para os profissionais e resultar em erros no preenchimento (28). 

A maioria das variáveis foi avaliada como excelente em relação à consistência. A data de notificação vs. a data de encerramento foi regular. O preenchimento correto desses campos é importante para verificar se a intervenção foi realizada em tempo oportuno (10). Desde 2015, todos os casos de violência notificados são encerrados no momento da notificação (10). A regular consistência dessa variável é passível de resolução, uma vez que as fichas devem ser submetidas à análise, sempre que possível, pelo Núcleo de Vigilância Epidemiológica da Unidade ou do nível municipal antes de serem encaminhadas para inclusão no Sistema (12). Uma medida para solucionar esse problema seria implementar o preenchimento automático do campo ou bloquear o salvamento da ficha caso as datas não correspondam, de forma a garantir a precisão e integridade dos dados registrados.

Entre as principais limitações deste estudo, reconhece-se que a inclusão de número maior de pontos temporais poderia ampliar a robustez das análises, no entanto a reformulação da ficha de notificação, em 2014 (20), inviabilizou a inclusão de dados de anos anteriores. O ano final, 2023, foi adotado por ser o período mais recente com dados consolidados no momento da pesquisa. Recomenda-se que estudos futuros explorem séries mais longas. A delimitação geográfica também é considerada uma limitação, assim sugere-se a replicação deste estudo em outras unidades federativas ou em âmbito nacional, possibilitando uma visão mais abrangente sobre a qualidade das notificações de automutilação entre adolescentes no Sinan. 

Outra limitação importante é a falta ou o preenchimento inadequado do campo aberto correspondente ao tipo de violência autoprovocada, pouco mais de 30,0%, acarretando um número elevado de notificações excluídas. Embora este estudo apresente boa avaliação das notificações analisadas, é importante considerar que os resultados poderiam apresentar variações caso fosse possível acessar a informação, referente ao tipo de violência autoprovocada, nas notificações excluídas. Ainda que se apresente algumas limitações, os achados não devem ser invalidados, pois são relevantes para a compreensão e o monitoramento da automutilação.

Para melhorar a qualidade dos dados de automutilação no Sinan, recomendam-se, em futuras atualizações da ficha de notificação, a inclusão de campo fechado com opções de preenchimento para tentativa de suicídio e automutilação e a ampliação no número de campos obrigatórios. Também se recomendam a educação continuada dos profissionais, principalmente daqueles que atuam em serviços que atendem adolescentes, para identificação, notificação e encaminhamento, assim como o monitoramento e a análise constante das notificações pelos órgãos responsáveis.

Os resultados deste estudo indicam níveis de completitude e consistência bons e excelentes, na maioria das variáveis, das notificações de automutilação por adolescentes no Sinan em Santa Catarina. Não foram identificadas duplicidades. A análise da tendência temporal revelou estabilidade na completitude e na consistência das informações no período estudado.
